# Incentive policy for the comprehensive development of young medical talents: an evolutionary game study

**DOI:** 10.3389/fpubh.2024.1325166

**Published:** 2024-02-02

**Authors:** Si Li, Lijuan He, Yaxin Huang, Dan Wang, Weihua Zhu, Zhisong Chen

**Affiliations:** ^1^Department of Personnel and Party Affairs, The First Affiliated Hospital with Nanjing Medical University, Nanjing, China; ^2^Business School, Nanjing Normal University, Nanjing, China; ^3^Stern School of Business, New York University, New York, NY, United States

**Keywords:** young medical talents, comprehensive development, evolutionary game, evolutionarily stable strategy, incentive policy

## Abstract

**Introduction:**

Currently in China, there is a lack of well-defined and viable incentive mechanisms at the governmental and hospital levels to support the development of young medical talents, thereby hindering their growth Existing studies primarily investigate the current state and trajectory of incentives, yet they inadequately address the distinctive characteristics of various stakeholders involved in medical talent incentive processes, particularly the lack of research on incentive mechanisms with Chinese attributes.

**Methods:**

This study adopts evolutionary game theory to investigate the dynamics of replication and the strategies for achieving evolutionary stability in the comprehensive development of young medical talents, considering both scenarios with and without supportive policies.

**Results:**

In the absence of any supportive policy measures, the evolutionarily stable strategy (ESS) point is *O*(0,0), the unstable equilibrium point is *C*(1,1), and the saddle points are *A*(0,1), *B*(1,0). The initial state of the system is at the unstable equilibrium point *C*(1,1), which means that the young medical talents and medical institutions adopt a combination of strategies (actively seeking comprehensive development and taking incentive measures). Under the scenario with supportive policies, the ESS point is *C*(1,1), the unstable equilibrium point is *O*(0,0), and the saddle points are *A*(0,1), *B*(1,0). The initial state of the system is at the unstable equilibrium point *O*(0,0), which means that young medical talents and medical institutions adopt (*N*,*N*) strategy combinations (inactively seeking comprehensive development, implementing no incentive measure).

**Discussion:**

(1) Government incentives play a crucial role in motivating young medical talents to seek comprehensive development. (2) The level of government incentive support for young medical talents should exceed the cost increment of individual efforts. Additionally, the policy support provided by the government to medical institutions should surpass the incentive support offered by these institutions to young medical talents. This will enhance the motivation and encouragement efforts of medical institutions in actively promoting comprehensive development among young medical talents. (3) With the backing of certain government incentive policies, medical institutions implementing incentive measures and young medical talents actively seeking comprehensive development will establish a virtuous cycle of mutual promotion.

## 1 Introduction

The young medical talents are the foundation and backbone of the hospital talent echelon, as well as an important new force for the development and construction of hospitals. The construction of young medical talents is an important foundation for guaranteeing the service quality and improving the service level of public hospitals (1). The “China’s 14th Five-Year Plan” for the Development of Health Talents proposes to build a highland of life and health talents, cultivating a group of innovative high-level talents, especially the young scientific and technological talents with multi-disciplinary background, as well as compound and innovative talents. Meanwhile, there is an ever-growing demand for high-quality medical services among the population. In the face of opportunities and challenges, it is crucial to train a group of young medical talents with comprehensive abilities who can adapt to the changing times.

Medical, teaching, and scientific research are known as the “three pillars” of high-quality hospital development. They represent the comprehensive strength of the hospitals and are essential for the comprehensive development of young medical talents. Young medical talents are in the stage of rapid accumulation of specialized knowledge and clinical experience, as well as in the golden period for improving their abilities and advancing their careers. Currently in China, the government does not have a perfect policy to support young medical talents. Due to the heavy workload in hospitals, there is a general tendency to prioritize utilization over cultivation. The specific and feasible incentive mechanism for this particular group has not been established at the government and hospital levels (2–4). From the perspective of talent echelon construction, young medical talents are the cornerstone of the development of medical institutions and the key to improving the medical level, and their career development and growth are related to the level of regional medical services. Based on the current situation, the development of young medical talents lacks mature incentive policy support, resulting in their lack of enthusiasm for work and lack of planning for career development, causing the unreasonable talent structure and layout of regional or medical institutions, and restricting the improvement of the overall medical level. Therefore, it is necessary to build a mature incentive mechanism for young talents.

According to a 2010 report by the World Health Organization, lack of motivation was identified as one of the top 10 major sources of inefficiency in health resources (5). The work efficiency of medical personnel, medical institutions, and the entire healthcare system will deteriorate in the absence of a sufficient number of motivated, operational, skilled, and trained medical workers. The national health system may not be fully functional, which could impact the safety and quality of medical and healthcare services (6, 7). Therefore, providing adequate incentives for medical personnel is of great significance in ensuring the safe and efficient operation of the healthcare system, as well as improving the overall health level of the population.

The China’s 14th Five-Year Plan for National Economic and Social Development and 2035 Long-term Goals Outline also emphasizes the implementation of a human resources-driven strategy to enhance the country’s strength. It highlights the need to improve talent evaluation and incentive mechanisms, comprehensively cultivate, attract, and effectively utilize talents, while fully harnessing their potential as a primary resource. The establishment of a modern hospital management system with Chinese characteristics is not only necessary to deepen the reform of public hospitals but also essential for promoting the reform of the medical and health system under the new normal. Improving the incentive system of medical talents has far-reaching significance for the development of modern hospitals. In developed countries, the development of human resources started early and was more systematic. In the hospital management, the human resource incentive theory is applied more mature, and some valuable incentive system design is put forward, comprehensively considering the quantity and quality of medical services.

Therefore, it is urgent to establish an incentive mechanism for young medical talents to promote the comprehensive improvement of the medical teaching and research ability of their groups. In this study, the theory of evolutionary game theory will be used to analyze the replication dynamics and evolutionary stability strategies of young medical talents and medical institutions under the support of government supporting policies, and try to reveal the evolution law and evolutionary stability strategies of the comprehensive development of young medical talents, so as to study the corresponding supporting policies of government departments on this basis. In this study, evolutionary game theory will be used to analyze the replication dynamics and evolutionary stability strategies of young medical talents and medical institutions under government support policies. The aim is to reveal the laws of evolution and stability strategies for comprehensive development of young medical talents, in order to inform corresponding government policies.

In the following sections, the corresponding literature review is first conducted in Section 2; the modeling symbols and assumptions of the replication dynamics and evolutionary stability strategies of young medical talents and medical institutions under the support of government supporting policies are defined in the beginning of section 3; then, in Section 3, decision models are built under two different scenarios based on game theory, including the evolutionary decision model without policy support (Section 3.1) and the evolutionary decision model with policy support (Section 3.2); the managerial insights and policy implications are discussed and summarized in Section 4; and the research contributions and foresights from this study are summarized and concluded in Section 5.

## 2 Literature review

### 2.1 Comprehensive development of medical talents

The rapid advancement of science and technology has transformed medicine from a simple natural discipline into a vast field that integrates various multidisciplinary technologies. In the era of rapid development in medical science and technology, along with the shift towards a modern bio-psycho-social medicine model, it is imperative for medical talents to possess comprehensive skills encompassing health professional knowledge, expertise in specific medical fields, and research capabilities to meet the evolving healthcare needs of humanity. Charles Boelen, the former director of WHO’s Health Talent Development Department (8), first proposed the training goal of “five-star doctors,” which includes health security providers, decision makers, health educators, community leaders and service managers. This proposal has received a strong response and widespread recognition from the global medical education community. The comprehensive development of medical talents has also received increasing attention from all sectors of society.

Many researchers have conducted in-depth studies on the comprehensive development of medical talents, which involve the training content, methods, and system as well as the construction of competency models for medical talents. Regarding the content of medical personnel training, the professional content and curriculum framework of medical talents in different disciplines are mostly analyzed from the perspective of a single specialty (9–13). For the training of medical professionals, more emphasis is placed on the teaching methods for physician skills (14–16) and effectiveness assessment (17, 18). Many academic organizations, such as medical specialty committees, publish industry guidelines that include the training content outline, training institutions, certification access, continuing education and other aspects for the doctor’s training system (19–21). Competency is a profound personal trait that distinguishes excellence in a job from ordinary individuals (22). Researchers have developed numerous influential clinical physician competency models, which are widely utilized in the training and education of medical professionals. These models can significantly enhance the quality of specialist training and yield positive outcomes (23–25).

Existing research on the comprehensive development of medical talent focuses primarily on learning and training itself, with few studies examining the motivation of medical talents to pursue comprehensive development and advancement. There is still a lack of effective countermeasures and suggestions on how to build a more mature incentive mechanism for medical talents, give full play to the subjective initiative of medical talents, and improve the quality and efficiency of learning and training.

### 2.2 Incentive mechanism for medical talents

Incentive refers to the means of mobilizing people’s enthusiasm and promoting behavior (26). The incentive mechanism is a standardized system arrangement and operational design for implementing incentive measures. Incentives refer to the comprehensive use of various incentive measures within an organizational system, which are established as fixed norms or systems and serve to motivate individuals towards achieving goals set by the organization (26, 27). A perfect medical talent incentive mechanism plays a vital role in the comprehensive development of doctors. The incentive mechanism often involves multiple subjects and objects with multi-interest relations, including the government, enterprises, individuals, and civil organizations.

The incentive plays a crucial role in fully utilizing the potential abilities of organizational members and enhancing the contribution and proficiency of medical talents. Numerous researchers have extensively investigated the role of medical talent incentives and contend that implementing appropriate incentive measures can enhance medical personnel’s sense of responsibility (28), work enthusiasm (29) and efficiency (30). Moreover, this approach can effectively mitigate burnout among healthcare staff while fostering their unwavering dedication to patient care, thereby continuously elevating the overall quality of medical services (2).

The role of incentive mechanism has been greatly valued by hospital managers and is widely used to attract and gather medical talents, encompassing both economic and non-economic incentives. In terms of economic incentives, the importance of salary and performance appraisal in motivating medical personnel has been widely acknowledged, with an underlying belief that increasing income can consistently foster their work enthusiasm (31, 32). Meanwhile, non-monetary economic incentives such as housing and transportation subsidies, along with retirement security provisions, can also serve as effective motivators for medical personnel (33, 34). Previous studies showed that certain non-economic interventions may serve as a more efficacious approach to enhancing medical staff motivation, necessitating the implementation of comprehensive strategies to augment the incentive effect (6), These encompass factors as fostering a conducive working atmosphere and promoting harmonious relationships (35, 36), ensuring clear delineation of job responsibilities (37), providing equitable opportunities for career advancement and professional growth (38), establishing rational and effective systems for rewards and penalties (32), intensifying medical service training programs (39), and cultivating positive hospital culture (40).

Researchers have adopted game theory to investigate the incentive mechanism, employing this method to examine the interaction between the structure of incentive mechanisms and strategize for optimizing incentives. The evolutionary game is a classic methodology for investigating trends in evolution, which could comprehensively consider multiple subject objects. It focuses on a group of participants with limited rationality as the research subject, incorporating dynamic analysis methods to incorporate factors influencing participant behavior into the model. This approach provides a systemic perspective for analyzing and understanding the evolution of group behavior. The evolutionary game is widely used in the design and optimization of incentive mechanisms. For example, Zheng et al. (41) constructed an evolutionary game model involving countries, universities, and researchers. Based on the evolutionary results of the model, a thorough analysis is conducted on the national incentives to researchers. Xie et al. (42) established an evolutionary game theory-based game model involving the government, farmers, and consumers to analyze the evolutionary process and stabilization strategies of key stakeholders in order to enhance safety management effectiveness. Sun et al. (43) constructed an evolutionary game model to explore cooperative optimization between healthcare and older adult social care organizations. However, there is a dearth of research on the design and optimization of incentive mechanisms for medical staff based on evolutionary game theory.

The existing researches on the incentive of medical talents mainly explore the current situation and path of incentives, but insufficiently pay attention to the characteristics of different stakeholders in the process of incentivizing medical talent. Therefore, it is impossible to combine the interaction of different stakeholders from the perspective of operational management.

### 2.3 Discussions

In conclusion, numerous researchers have explored the comprehensive development of young medical talents and the incentive mechanisms pertaining to their field. The researches on the comprehensive development of medical talents pay more attention to the learning and training itself. The researches on the incentive mechanism of medical talents mainly explores the current situation and the path of incentive. Based on the analysis of relevant researches, the existing studies do not fully consider the following key issues: (i) Insufficient attention towards the motivation of medical professionals in pursuing comprehensive development and advancement. (ii) Insufficient attention to the characteristics of different stakeholders in the medical talent incentive process. (iii) Insufficient attention to the incentive mechanism for medical professionals in accordance with the Chinese healthcare system. (iv) More focus was played to the effectiveness of incentive mechanism on the individual level, insufficient attention was played to the effectiveness of incentive mechanism on the government and hospital management level.

In China, medical and health institutions are dominated by public medical and health institutions. It has an overall layout with non-profit medical institutions as the main body and for-profit medical institutions as the supplement (44). Public hospitals refer to hospitals that are wholly owned or controlled by state-owned capital in the capital structure of hospitals. The fundamental characteristics of public hospitals are to represent the interests of state-owned capital, possess a nature of public welfare, offer essential medical services, and undertake the social responsibility of ensuring health equity (45). Currently, China’s public hospitals boast the most comprehensive supporting facilities nationwide, attracting the finest medical professionals in the country (46, 47). Most of the Chinese medical professionals work within the public healthcare system, and their growth is closely linked to various government policies. Therefore, it is necessary to explore the incentive mechanism for medical talents from the perspective of supporting government policies and the perspective of medical institution incentive, and give full play to the subjective initiative of medical talents.

## 3 Model formulation and analysis

### 3.1 Model assumptions and parameters setting

To analyze the replication dynamics and evolutionary stability strategies between medical institutions and young talents from an evolutionary game perspective, we first define the strategies of both sides. The strategies employed by medical institutions primarily encompass implementing incentive measures (A) and implementing no incentive measure (N). The strategies of young medical talents mainly consist of actively seeking comprehensive development (A) and inactively seeking comprehensive development (N).

For modeling needs, this article makes the following model assumptions and parameter settings. The effort cost incurred by young medical talents when they actively seek comprehensive development (A) is $C_{H}$ , and the effort cost incurred by inactively seeking comprehensive development is $C_{L}$ ; regardless of whether young medical talents actively seek comprehensive development or not, medical institutions will provide young medical talents with regular salary S ; the contribution that young medical talents can bring to medical institutions by actively seeking comprehensive development (A) is B . On the contrary, if young medical talents inactively seek comprehensive development (N), they can only bring B−K contributions to medical institutions. 𝐾 refers to the losses caused to medical institutions because young medical talents inactively seek comprehensive development. Medical institutions may take incentive measures (A) to encourage young medical talents to actively seek comprehensive development, and provide young talents with incentive subsidies of R ; conversely, if medical institutions do not take incentive measures (N), they will not provide corresponding incentive subsidies to young talents. In order to encourage young talents to take the initiative to seek comprehensive development, government departments provide supporting subsidies $S_{E}$ for their active pursuit of comprehensive development. At the same time, in order to encourage medical institutions to adopt incentives to encourage young talents to actively seek comprehensive development, they adopt incentive measures and supporting subsidies $S_{M}$ for their actions. In order to ensure that the model has practical significance and value, it is assumed that $S>C_{H}>C_{L}$ , B>S+K+R .

### 3.2 Evolutionary game model under the scenario without supportive policies

In the absence of supportive policies, the utility functions of medical institutions and young medical talents under different strategy combinations, and the corresponding evolutionary game payoff matrix between young medical talents and medical institutions can be depicted as follows (as shown in Table 1).

**Table 1:** The payoff matrix for the evolutionary game under the scenario without supportive policies.

	Implementing incentive measures (A) *y*	Implementing no incentive measure (N) 1 – *y*
Actively seeking comprehensive development (A) *x*	$\left(\Pi_{E}^{\mathrm{AA}}=S+R-C_{H},\Pi_{M}^{\mathrm{AA}}=B-S-R\right)$	$\left(\Pi_{E}^{\mathrm{AN}}=S-C_{H},\Pi_{M}^{\mathrm{AN}}=B-S\right)$
Inactively seeking comprehensive development (N) 1 – *x*	$\left(\Pi_{E}^{NA}=S+R-C_{L},\Pi_{M}^{NA}=B-S-K-R\right)$	$\left(\Pi_{E}^{NN}=S-C_{L},\Pi_{M}^{NN}=B-S-K\right)$

In the game of the comprehensive development of young medical talents, we assume that the proportion of young medical talents who take the initiative to seek a comprehensive development strategy is x , and the proportion of the young medical talents who inactively seek comprehensive development (N) strategy is 1−x . Moreover, in the medical institution group, we assume that the proportion of medical institutions that implement the incentive measures (A) is y , the proportion of medical institutions that do not implement the incentive measures (N) is 1−y .

Therefore, the anticipated benefits and the average anticipated benefits of young medical talents who actively seek comprehensive development (A) strategy and those who inactively seek comprehensive development (N) can be expressed as the following Equations 1–3:


(1) $$\Pi_{E}^{A}=y\Pi_{E}^{\mathrm{AA}}+\left(1-y\right)\Pi_{E}^{\mathrm{AN}}=S+yR-C_{H}$$

(2) $$\Pi_{E}^{N}=y\Pi_{E}^{NA}+\left(1-y\right)\Pi_{E}^{NN}=S+yR-C_{L}$$

(3) $$\Pi_{E}=x\Pi_{E}^{A}+\left(1-x\right)\Pi_{E}^{N}=S+yR-xC_{H}-\left(1-x\right)C_{L}$$

Similarly, the anticipated benefits and average anticipated benefits of implementing incentive measures (A) and implementing incentive measures (N) can be expressed as the following Equations 4–6:


(4) $$\Pi_{M}^{A}=x\Pi_{M}^{\mathrm{AA}}+\left(1-x\right)\Pi_{M}^{NA}=\left(B-S-R\right)-\left(1-x\right)K$$

(5) $$\Pi_{M}^{N}=x\Pi_{M}^{\mathrm{AN}}+\left(1-x\right)\Pi_{M}^{NN}=B-S-\left(1-x\right)K$$

(6) $$\Pi_{M}=y\Pi_{M}^{A}+\left(1-y\right)\Pi_{M}^{N}=B-S-\left(1-x\right)K-yR$$

**Figure 1:**
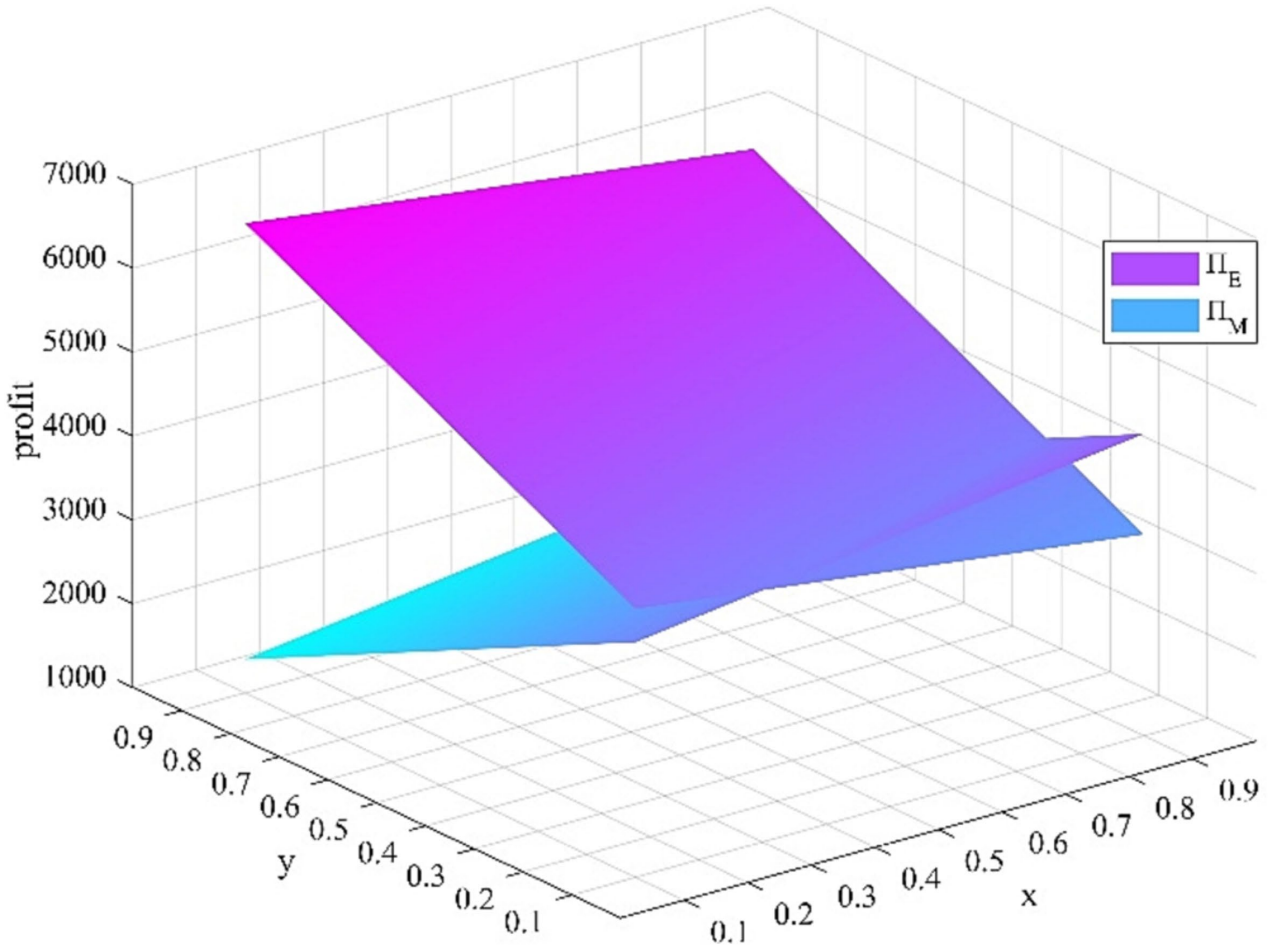
Impact of x and y change.

Without loss of generality, the corresponding parameters can be set as follows: B=10000 , S=5000 , R=3000 , K=1000 , $C_{H}=2000$ , $C_{L}=1000$ . On this basis, we can investigate how the changes of the proportion of young medical talents who take the initiative to seek a comprehensive development strategy x and the proportion of medical institutions that implement the incentive measures y , impact the anticipated benefits for the young medical talents and the medical institutions. The change range of x and y are all be located in $\left[0,1\right]$ . The sensitivity analysis results of x and y is shown in Figure 1. As the proportion of young medical talents who take the initiative to seek a comprehensive development strategy x increases, the anticipated benefit of the young medical talents decreases, the anticipated benefit of the medical institutions increases. As the proportion of medical institutions that implement the incentive measures y increases, the anticipated benefit of the young medical talents increases, the anticipated benefit of the medical institutions decreases.

#### 3.2.1 Evolutionary game model and its solution

Therefore, the replication dynamic equation for young medical talents to actively seek comprehensive development (A) strategy is expressed as the following equation:


$$\frac{dx}{dt}=x\left(\Pi_{E}^{A}-\Pi_{E}\right)=-x\left(1-x\right)\left(C_{H}-C_{L}\right)$$

Similarly, the replication dynamic equation for medical institutions to implement incentive measures (A) is expressed as the following equation:


$$\frac{\mathrm{dy}}{dt}=y\left(\Pi_{M}^{A}-\Pi_{M}\right)=-y\left(1-y\right)R$$

We define that $F\left(x,y\right)=\frac{dx}{dt}=0$ , $G\left(y,x\right)=\frac{\mathrm{dy}}{dt}=0$ . Then the two-dimensional continuous dynamic system has four local equilibrium points $\left(x,y\right)=\left\{\left(0,0\right),\left(0,1\right),\left(1,0\right),\left(1,1\right)\right\}$ in the plane $\left\{\left(x,y\right):0\leq x,y\leq 1\right\}$ .

The stability of the equilibrium point for a group dynamic described by a system of differential equations is determined through local stability analysis of the Jacobi matrix derived from the system (18–21). According to Friedman’s proposed method, the stability of the equilibrium point can be determined through local stability analysis of the system’s Jacobi matrix, in conjunction with Equation (11) and (12). Therefore, the Jacobi matrix and the corresponding determinant and its trace of the system can be obtained, respectively.


$$J=\left[\begin{array}{cc} \frac{\partial F\left(x,y\right)}{\partial x} & \frac{\partial F\left(x,y\right)}{\partial y}\\ \frac{\partial G\left(y,x\right)}{\partial x} & \frac{\partial G\left(y,x\right)}{\partial y} \end{array}\right]=\left[\begin{array}{cc} -\left(1-2x\right)\left(C_{H}-C_{L}\right) & 0\\ 0 & -\left(1-2y\right)R \end{array}\right]\text{,}$$

$$\det\left(J\right)=\left(1-2x\right)\left(C_{H}-C_{L}\right)\left(1-2y\right)R\text{,}$$

$$Tr\left(J\right)=-\left(1-2x\right)\left(C_{H}-C_{L}\right)-\left(1-2y\right)R\text{.}$$

The stability analysis of four equilibrium points was conducted based on local stability analysis, and the corresponding results are presented in Table 2. The analysis reveals that among the four local equilibrium points, one exhibits an evolutionarily stable strategy, while another is characterized as an unstable equilibrium point, with the remaining two being saddle points.

**Table 2:** Results of the local stability analysis.

Equilibrium points	Determinant *Det(J)*	*Det(J)* symbol	Trace *Tr(J)*	*Tr(J)* symbol	Stability
$O\left(0,0\right)$	$\det\left(0,0\right)=\left(C_{H}-C_{L}\right)R$	+	$Tr\left(0,0\right)=-\left(C_{H}-C_{L}\right)-R$	−	ESS
$A\left(0,1\right)$	$\det\left(0,1\right)=-\left(C_{H}-C_{L}\right)R$	−	$Tr\left(0,1\right)=-\left(C_{H}-C_{L}\right)+R$	Indeterminacy	Saddle point
$B\left(1,0\right)$	$\det\left(1,0\right)=-\left(C_{H}-C_{L}\right)R$	−	$Tr\left(1,0\right)=\left(C_{H}-C_{L}\right)-R$	Indeterminacy	Saddle point
$C\left(1,1\right)$	$\det\left(1,1\right)=\left(C_{H}-C_{L}\right)R$	+	$Tr\left(1,1\right)=\left(C_{H}-C_{L}\right)+R$	+	Instability

#### 3.2.2 Evolutionary phase diagram and evolutionary path

**Figure 2:**
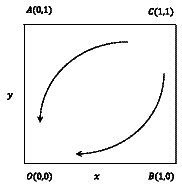
Replication dynamics and stability of young medical talents and medical institutions.

The replication dynamics and stability of the evolutionary game group consisting of young medical talents and medical institutions in the absence of any supportive policy measures are showed in Figure 2. The evolutionarily stable strategy (ESS) point is $O\left(0,0\right)$ , the unstable equilibrium point is $C\left(1,1\right)$ , and the saddle points are $A\left(0,1\right)$ , $B\left(1,0\right)$ . The initial state of the system is at the unstable equilibrium point $C\left(1,1\right)$ , which means that the young medical talents and medical institutions adopt a combination of strategies (actively seeking comprehensive development and taking incentive measures). The system will gradually evolve from the unstable equilibrium point $C\left(1,1\right)$ to the ESS point $O\left(0,0\right)$ , which means that young medical talents and medical institutions adopt $\left(N,N\right)$ strategy combinations (inactively seeking comprehensive development, no incentive measure). Clearly, in the absence of supportive policies, young medical talents lack sufficient economic incentives to actively seek comprehensive development, while medical institutions lack adequate motivation to implement incentive measures.

### 3.3 Evolutionary game model under the scenario with supportive policies

In the context of supporting policies, the utility functions of medical institutions and young medical talents under different strategy combinations, and the corresponding evolutionary game payoff matrix between young medical talents and medical institutions can be depicted as follows (as shown in Table 3).

**Table 3:** The payoff matrix for the evolutionary game under the scenario without supportive policies.

Young medical talent	Implementing incentive measures (A) *y*	Implementing no incentive measure (N) 1 – *y*
Actively seeking comprehensive development (A) *x*	$\left(\Pi_{E}^{\mathrm{AA}\prime }=S+S_{E}+R-C_{H},\Pi_{M}^{AA^{\prime }}=B-S+S_{M}-R\right)$	$\left(\Pi_{E}^{\mathrm{AN}\prime }=S+S_{E}-C_{H},\Pi_{M}^{AN^{\prime }}=B-S\right)$
Inactively seeking comprehensive development (N) 1 – *x*	$\left(\Pi_{E}^{NA\prime }=S+R-C_{L},\Pi_{M}^{NA^{\prime }}=B-S+S_{M}-K-R\right)$	$\left(\Pi_{E}^{NN\prime }=S-C_{L},\Pi_{M}^{NN^{\prime }}=B-S-K\right)$

In the game of the comprehensive development of young medical talents, we assume that the proportion of young medical talents who actively seek comprehensive development strategy (A) is x , and the proportion of the young medical talents who inactively seek comprehensive development (N) strategy is 1−x . Moreover, in the medical institution group, we assume that the proportion of medical institutions that implement incentive measures (A) is y , the proportion of medical institutions that implement no incentive measure (N) is 1−y .

Therefore, the anticipated benefits and the average anticipated benefits of young medical talents who actively seek comprehensive development (A) strategy and those who inactively seek comprehensive development can be expressed as the following Equations 7–9:


(7) $$\Pi_{E}^{A\prime }=y\Pi_{E}^{\mathrm{AA}\prime }+\left(1-y\right)\Pi_{E}^{\mathrm{AN}\prime }=S+S_{E}+yR-C_{H}$$

(8) $$\Pi_{E}^{N\prime }=y\Pi_{E}^{NA\prime }+\left(1-y\right)\Pi_{E}^{NN\prime }=S+yR-C_{L}$$

(9) $$\Pi_{E}^{\prime }=x\Pi_{E}^{A\prime }+\left(1-x\right)\Pi_{E}^{N\prime }=S+xS_{E}+yR-xC_{H}-\left(1-x\right)C_{L}$$

Similarly, the anticipated benefits and average anticipated benefits of implementing incentive measures (A) and implementing incentive measures (N) strategies can be expressed as the following Equations 10–12:


(10) $$\Pi_{M}^{A\prime }=x\Pi_{M}^{\mathrm{AA}\prime }+\left(1-x\right)\Pi_{M}^{NA\prime }=\left(B-S+S_{M}-R\right)-\left(1-x\right)K$$

(11) $$\Pi_{M}^{N\prime }=x\Pi_{M}^{\mathrm{AN}\prime }+\left(1-x\right)\Pi_{M}^{NN\prime }=B-S-\left(1-x\right)K$$

(12) $$\Pi_{M}^{\prime }=y\Pi_{M}^{A\prime }+\left(1-y\right)\Pi_{M}^{N\prime }=B-S-\left(1-x\right)K+yS_{M}-yR$$

**Figure 3:**
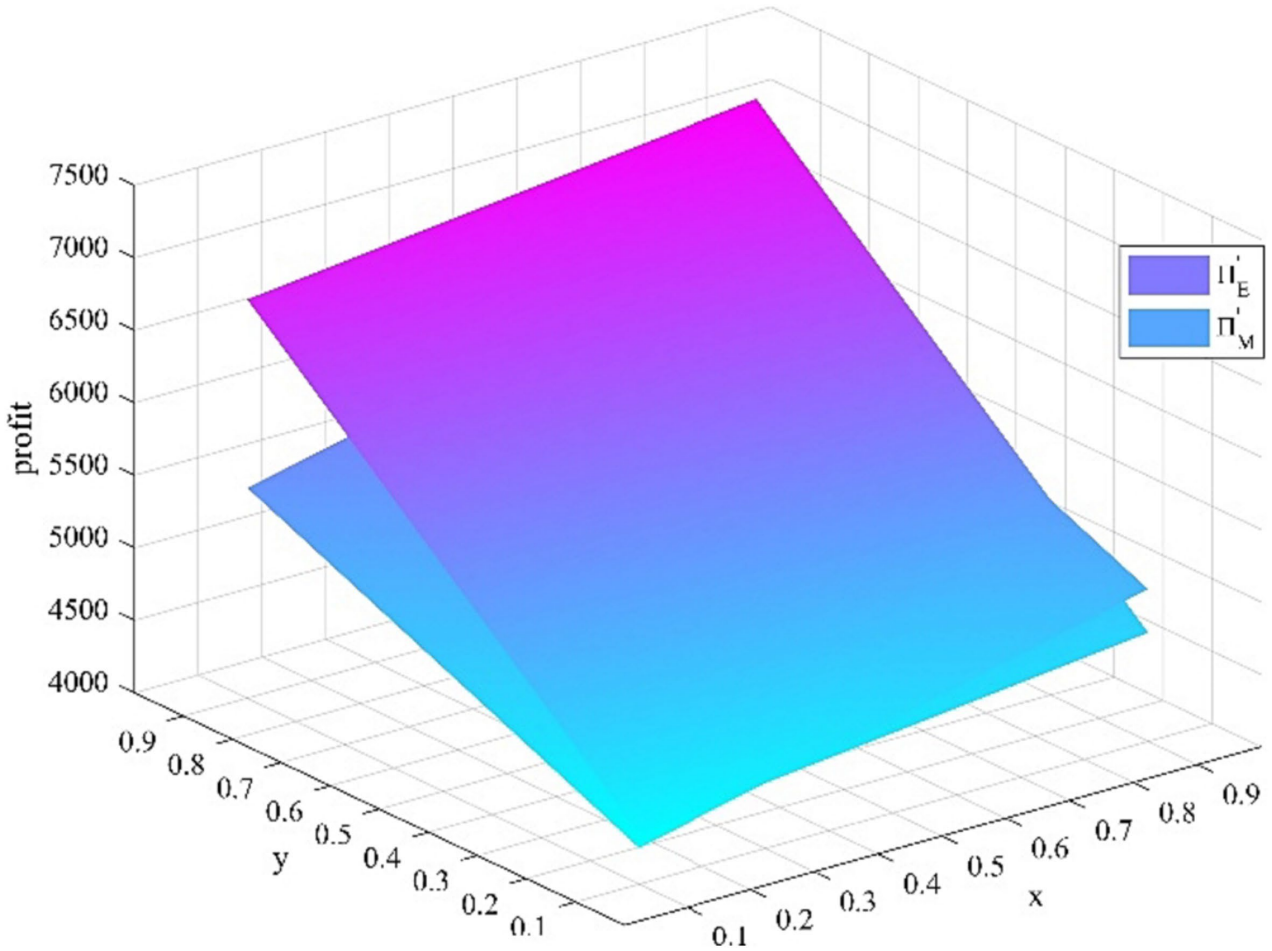
Impact of x and y change.

On the basis of parameters setting in section 3.2, the corresponding parameters can be set as follows: $S_{M}=4500$ , $S_{E}=1500$ . Likewise, we can investigate how the changes of x and y impact the anticipated benefits for the young medical talents and the medical institutions. The change range of x and y are all be located in $\left[0,1\right]$ . The sensitivity analysis results of x and y is shown in Figure 3. As the proportion of young medical talents who take the initiative to seek a comprehensive development strategy x increases, the anticipated benefit of the young medical talents increases, the anticipated benefit of the medical institutions increases. As the proportion of medical institutions that implement the incentive measures y increases, the anticipated benefit of the young medical talents increases, the anticipated benefit of the medical institutions increases.

#### 3.3.1 Evolutionary game model and its solution

Therefore, the replication dynamic equation for young medical talents to actively seek comprehensive development (A) strategy is expressed as the following equation:


$$\frac{dx}{dt}=x\left(\Pi_{E}^{A\prime }-\Pi_{E}^{\prime }\right)=x\left(1-x\right)\left[S_{E}-\left(C_{H}-C_{L}\right)\right]$$

Similarly, the replication dynamic equation for medical institutions to implement incentive measures (A) is expressed as the following equation:


$$\frac{\mathrm{dy}}{dt}=y\left(\Pi_{M}^{A\prime }-\Pi_{M}^{\prime }\right)=y\left(1-y\right)\left(S_{M}-R\right)$$

We define that $F\left(x,y\right)=\frac{dx}{dt}=0$ , $G\left(y,x\right)=\frac{\mathrm{dy}}{dt}=0$ . Then the two-dimensional continuous dynamic system has four local equilibrium points $\left(x,y\right)=\left\{\left(0,0\right),\left(0,1\right),\left(1,0\right),\left(1,1\right)\right\}$ in the plane $\left\{\left(x,y\right):0\leq x,y\leq 1\right\}$ .

**Figure 4:**
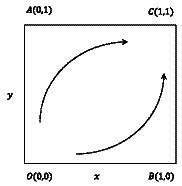
Replication dynamics and stability of young medical talents and medical institutions.

The stability of the equilibrium point for a group dynamic described by a system of differential equations is determined through local stability analysis of the Jacobi matrix derived from the system (18–21). According to Friedman’s proposed method, the stability of the equilibrium point can be determined through local stability analysis of the system’s Jacobi matrix, in conjunction with Equation (11) and (12). Therefore, the Jacobi matrix and the corresponding determinant and its trace of the system can be obtained, respectively (Figure 4).


$$\begin{array}{c} J=\left[\begin{array}{cc} \frac{\partial F\left(x,y\right)}{\partial x} & \frac{\partial F\left(x,y\right)}{\partial y}\\ \frac{\partial G\left(y,x\right)}{\partial x} & \frac{\partial G\left(y,x\right)}{\partial y} \end{array}\right]\\ =\left[\begin{array}{cc} \left(1-2x\right)\left[S_{E}-\left(C_{H}-C_{L}\right)\right] & 0\\ 0 & \left(1-2y\right)\left(S_{M}-R\right) \end{array}\right]\text{,} \end{array}$$

$$\det\left(J\right)=\left(1-2x\right)\left[S_{E}-\left(C_{H}-C_{L}\right)\right]\left(1-2y\right)\left(S_{M}-R\right)\text{,}$$

$$Tr\left(J\right)=\left(1-2x\right)\left[S_{E}-\left(C_{H}-C_{L}\right)\right]+\left(1-2y\right)\left(S_{M}-R\right)\text{.}$$

The stability analysis of four equilibrium points was conducted based on local stability analysis, and the corresponding results are presented in Table 4. In the context of supporting policies, when the government supporting subsidies for young medical talents actively seeking comprehensive development and medical institutions implement incentive measures meet the condition $S_{E}>C_{H}-C_{L}$ and $S_{M}>R$ , the analysis reveals that among the four local equilibrium points, one exhibits an evolutionarily stable strategy, while another is characterized as an unstable equilibrium point, with the remaining two being saddle points.

**Table 4:** Results of the local stability analysis.

Equilibrium Points	Determinant *Det(J)*	*Det(J)* symbol	Trace *Tr(J)*	*Tr(J)* symbol	Stability
$O\left(0,0\right)$	$\det\left(0,0\right)=\left[S_{E}-\left(C_{H}-C_{L}\right)\right]\left(S_{M}-R\right)$	+	$Tr\left(0,0\right)=\left[S_{E}-\left(C_{H}-C_{L}\right)\right]+\left(S_{M}-R\right)$	+	Instability
$A\left(0,1\right)$	$\det\left(0,1\right)=-\left[S_{E}-\left(C_{H}-C_{L}\right)\right]\left(S_{M}-R\right)$	−	$Tr\left(0,1\right)=\left[S_{E}-\left(C_{H}-C_{L}\right)\right]-\left(S_{M}-R\right)$	Indeterminacy	Saddle point
$B\left(1,0\right)$	$\det\left(1,0\right)=-\left[S_{E}-\left(C_{H}-C_{L}\right)\right]\left(S_{M}-R\right)$	−	$Tr\left(1,0\right)=-\left[S_{E}-\left(C_{H}-C_{L}\right)\right]+\left(S_{M}-R\right)$	Indeterminacy	Saddle point
$C\left(1,1\right)$	$\det\left(1,1\right)=\left[S_{E}-\left(C_{H}-C_{L}\right)\right]\left(S_{M}-R\right)$	+	$Tr\left(1,1\right)=-\left[S_{E}-\left(C_{H}-C_{L}\right)\right]-\left(S_{M}-R\right)$	−	ESS

#### 3.3.2 Evolutionary phase diagram and evolutionary path

The replication dynamics and stability of the evolutionary game group consisting of young medical talents and medical institutions with supportive policy measures are showed in Figure 4. The ESS point is $C\left(1,1\right)$ , the unstable equilibrium point is $O\left(0,0\right)$ , and the saddle points are $A\left(0,1\right)$ , $B\left(1,0\right)$ . The initial state of the system is at the unstable equilibrium point $O\left(0,0\right)$ , which means that young medical talents and medical institutions adopt $\left(N,N\right)$ strategy combinations (inactively seeking comprehensive development, implementing no incentive measure). The system will gradually evolve from the unstable equilibrium point $O\left(0,0\right)$ to the ESS point $C\left(1,1\right)$ , which means that the young medical talents and medical institutions adopt a combination of strategies (actively seeking comprehensive development and implementing incentive measures).

Obviously, in the context of supporting policies, when the government supporting subsidies for young medical talents actively seeking the comprehensive development and medical institutions taking incentive measures meet the condition $S_{E}>C_{H}-C_{L}$ and $S_{M}>R$ , young medical talents are sufficiently driven by economic incentives to actively seek comprehensive development, while medical institutions possess adequate motivation to implement incentive measures.

## 4 Managerial insights and policy implications

Through modeling and numerical analysis, this study examines the replication dynamics and evolutionary stability strategies for cultivating young medical talents in both supportive and non-supportive policy contexts. The management insights and policy implications that are derived from the modelling analysis can be summarized as follows:

1. Only when supported by policies, medical institutions will take incentive measures, and young medical talents will actively seek comprehensive development. Government incentives play a crucial role in motivating young medical talents to seek comprehensive development. The attribute of public welfare is inherent in public hospitals, which categorizes them as providers of public services and institutions. Therefore, the reform of the public hospital incentive system is closely correlated with the guidance and support provided by the government. The Opinions on Deepening the Reform of the Medical and Health System were issued by China in 2009, and since then, the new medical reform has been gradually deepened. This poses a great challenge to public hospitals, inevitably leading to numerous obstacles in this process. In order to ensure the smooth implementation of the public hospital reform, government departments need to provide strong policy support for the incentive system reform of public hospitals at an overall level. Firstly, the government needs to introduce relevant policies to guide the reform of the incentive system in public hospitals, clarifying the direction and content of reform, as well as the methods and approaches for implementing it. Secondly, incentives are generally realized through material rewards and non-material incentives, and the incentive system reform will inevitably increase the expenditure pressure of public hospitals. Public hospitals are government-funded non-profit institutions. Therefore, the government needs to provide economic and policy support in order to help public hospitals promote the reform of their incentive system and maximize its effectiveness.
2. The government’s policy support level needs to reach a certain threshold. Specifically, the incentive support for young medical talents should exceed the cost increment of individual efforts ( $S_{E}>C_{H}-C_{L}$ ). Additionally, the policy support for medical institutions should surpass the incentive support provided by these medical institutions to young medical talents ( $S_{M}>R$ ). This will enhance the efforts of medical institutions in motivating and encouraging young medical talents to actively seek comprehensive development. The most typical representatives of knowledge-based employees, medical talents have a high educational background and personal accomplishments. They also have certain particularities in terms of psychological needs, working methods, personal characteristics, and values. Compared with the general staff, not only are they limited to the most basic physiological and material needs, but also higher-level needs. The government should closely align with the needs of medical professionals, starting from a humanistic perspective. They should involve medical experts in the development of incentive systems and formulate effective policies that cater to their requirements. The government should guide medical institutions to promote performance reform, innovate performance accounting methods, and fully unleash the subjective initiative of medical talents in accordance with the principle of equitable compensation based on workload. The government should concurrently provide adequate financial compensation to public hospitals and enhance support policies, in order to alleviate the burden of public hospital management and effectively invigorate them. On one hand, it enables public hospitals to effectively leverage their inherent characteristics for social welfare, on the other hand, it also allows adequate space for medical personnel to receive material incentives.
3. With the support of government incentive policies, medical institutions implementing incentive measures and young medical talents actively seeking comprehensive development will establish a virtuous cycle of mutual promotion [adopt $\left(A,A\right)$ strategy combination]. Under the context of supporting policies, the comprehensive development of medical talents benefits the improvement of the platform of medical institutions and enhances the level of comprehensive diagnosis and treatment. Meanwhile, a reasonable incentive mechanism for medical institutions promotes the comprehensive development of medical talents, thereby complementing each other. It can be observed that medical institutions and medical professionals share common objectives. Only by striving to establish a mutually beneficial incentive mechanism between medical institutions and medical talents can incentive policies truly achieve their maximum impact. The establishment and implementation of a medical talent incentive mechanism is a systematic and lengthy process. In order to build a diversified medical talent incentive system, the government and medical institutions should not only optimize the salary incentive mechanism, but also build an efficient medical talent training system, strengthen talent policy support, increase the proportion of non-monetary incentive, and constantly improve the diversity and precision of post incentive. Meanwhile, with government policy support, it is necessary for medical institutions to establish an evaluation system to assess the operational effectiveness of incentive mechanisms. This system should include professional evaluations, tracking feedback and adjustments, continuous optimization, in order to promote a positive development cycle between medical institutions and healthcare professionals.

## 5 Conclusion

Since existing studies primarily investigate the current state and trajectory of incentives, they inadequately address the distinctive characteristics of various stakeholders involved in medical talent incentive processes, particularly the lack of research on incentive mechanisms with Chinese attributes. Our study tries to apply evolutionary game theory to investigate the dynamics of replication and the strategies for achieving evolutionary stability in the comprehensive development of young medical talents, considering both scenarios with and without government incentive policies. The corresponding management insights and policy implications are discussed and summarized based on this foundation. The analysis results indicate as follows: (1) Government incentives play a crucial role in motivating young medical talents to seek comprehensive development. (2) The level of government incentive support for young medical talents should exceed the cost increment of individual efforts. Additionally, the policy support provided by the government to medical institutions should surpass the incentive support offered by these institutions to young medical talents. This will enhance the motivation and encouragement efforts of medical institutions in actively promoting comprehensive development among young medical talents. (3) With the support of government incentive policies, medical institutions implementing incentive measures and young medical talents actively seeking comprehensive development will establish a virtuous cycle of mutual promotion.

The study holds significant value for both theoretical and practical realms. Theoretical significance arises from the application of game theory principles to formulate a rational incentive mechanism model for medical talents. This model is designed in collaboration with relevant government departments and medical institutions, amalgamating the interests of various stakeholders. The focus is on evaluating the impact of the incentive mechanism at the governmental and hospital management levels. By doing so, the research contributes to the enrichment of the conceptual framework in medical talent incentive studies, broadening the overall research perspective. On the practical front, the study delves into the realm of government policy support and incentives within medical institutions. It establishes a diversified incentive model that incorporates the active participation of the government, medical institutions, and medical talents. The objective is to explore an effective incentive mechanism for medical professionals. This model, as developed in the study, stands as a valuable reference for decision-making processes within relevant government departments and medical institutions when formulating policies related to medical talent incentives. In essence, the research not only advances theoretical understanding by incorporating game theory into the incentive model but also provides tangible and applicable insights for policymakers and medical institutions. It is poised to serve as a foundation for the development and enhancement of effective medical talent incentive policies.

Due to a shortage of pertinent literature, constrained research funding, and challenges in acquiring empirical data, this study predominantly concentrates on the theoretical exploration of the incentive evolutionary mechanism and its accompanying policies to foster the holistic development of young medical talent. Despite unearthing insightful findings, certain crucial research aspects merit further investigation in future studies. First, the evolutionary game model can be extended to be three-agent evolutionary game model, incorporating the government support strategies and behaviors into the game model for comprehensive analysis, in our future research. Second, some empirical data regarding the comprehensive development of young medical talent can be collected and simulated to verify the analytical results of evolutionary game model in our future research. Third, some typical cases regarding the comprehensive development of young medical talent can be excavated to verify the analytical results of evolutionary game model and strengthen the effectiveness of the corresponding supportive policies in our future research.

## Data availability statement

The original contributions presented in the study are included in the article/supplementary material, further inquiries can be directed to the corresponding authors.

## Author contributions

SL: Conceptualization, Methodology, Writing – original draft, Writing – review & editing. LH: Writing – original draft. YH: Writing – review & editing. DW: Writing – original draft. WZ: Conceptualization, Writing – review & editing. ZC: Conceptualization, Methodology, Writing – original draft, Writing – review & editing.
